# Ethoxylated Butoxyethanol-BADGE Adducts—New Potential Migrants from Epoxy Resin Can Coating Material

**DOI:** 10.3390/ma14133682

**Published:** 2021-07-01

**Authors:** Monika Beszterda, Małgorzata Kasperkowiak, Magdalena Frańska, Sandra Jęziołowska, Rafał Frański

**Affiliations:** 1 Department of Food Biochemistry and Analysis, Poznań University of Life Sciences, Mazowiecka 48, 60-623 Poznań, Poland; monika.beszterda@gmail.com; 2 Center for Advanced Technology, Adam Mickiewicz University, Uniwersytetu Poznańskiego 10, 61-614 Poznań, Poland; malgorzata.kasperkowiak@amu.edu.pl; 3 Institute of Chemistry and Technical Electrochemistry, Poznań University of Technology, Berdychowo 4, 60-965 Poznań, Poland; magdalena.franska@put.poznan.pl; 4 Faculty of Chemistry, Adam Mickiewicz University, Uniwersytetu Poznańskiego 8, 61-614 Poznań, Poland; sanjez@st.amu.edu.pl

**Keywords:** epoxy resin, can coating, mass spectrometry, liquid chromatography, bisphenol A diglycidyl ether, buthoxyethanol, ethoxylates

## Abstract

The acetonitrile extracts of can-coating materials have been analyzed by using high-pressure liquid chromatography/electrospray ionization-mass spectrometry (HPLC/ESI-MS). On the basis of detected ions [M + H]^+^, [M + NH_4_]^+^, [M + Na]^+^ and product ions, the ethoxylated butoxyethanol-bisphenol A diglycidyl ether adducts were identified in two of the analyzed extracts. Although the oxyethylene unit-containing compounds are widely used for the production of different kinds of materials, the ethoxylated species have not been earlier detected in epoxy resin can-coatings.

## 1. Introduction

Epoxy resins are the materials most commonly used as internal lacquers for direct food contact in food cans. They are usually formed by the condensation of epichlorohydrin and bisphenol A (BPA), followed by dehydrohalogenation, which yields bisphenol A diglycidyl ethers (BADGEs) of varying degrees of condensation (the smallest product of this reaction is BADGE). In order to obtain the desired physicochemical properties of epoxy resins, a number of compounds are used that act as curing agents, crosslinking agents, lubricating agents, hardeners, diluents, fillers, pigments, plasticizers, accelerators, etc. Due to by-product formations, incomplete polymerization, degradation processes, etc., the obtained epoxy resins are not pure compounds and may contain contaminants that can easily migrate into the surrounding medium, such as food or beverages [1,2,3]. This problem has prompted a vast number of studies devoted to the analysis of migrants (mainly BPA, BADGE, and its conjugates), which are present in the food-contact can-coatings [3,4,5,6,7,8,9,10,11,12,13,14,15,16].

**Scheme 1:**
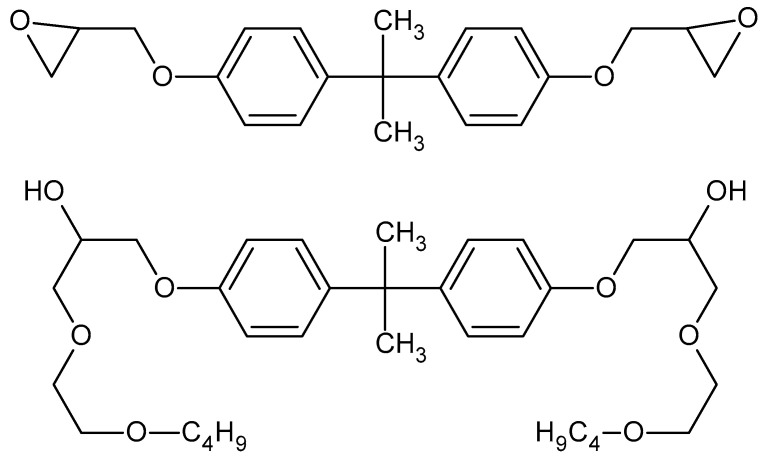
Structures of BADGE and BADGE + 2BuOEtOH.

Among miscellaneous BADGE conjugates, the adducts of BADGE with butoxyethanol (BuOEtOH) have been detected in the extracts of can-coating material, e.g., BADGE + BuOEtOH, BADGE + 2BuOEtOH, BADGE + BuOEtOH + H_2_O, and a few others [15,16,17]. The structure of BADGE and plausible structure of BADGE + 2BuOEtOH are shown in Scheme 1 (the formation of BADGE + 2BuOEtOH regioisomers is possible, [17]).

Butoxyethanol is one of the smallest surfactants, and it is a very common solvent used in many domestic and industrial products. Therefore it has attracted attention as a compound to which people can be exposed to [18,19,20,21]. Butoxyethanol is usually produced by the ethoxylation reaction of butanol with ethylene oxide. Therefore, the by-products can be formed during its production, namely compounds that contain more oxyethylene units, thus BuO(EtO)_n_H (EtO stands for the oxyethylene unit CH_2_CH_2_O).

In this short communication, we report the HPLC/ESI-MS detection of the adducts of BADGE with ethoxylated butoxyethanols, thus BADGE + BuO(EtO)_n_H + BuO(EtO)_m_H in the extracts of can-coating material. To the best of our knowledge, these kind of adducts have not been detected yet in such extracts.

## 2. Materials and Methods

A total number of 10 canned foods, such as meat products (3), seafood in oil (3), plant products (3), newborn and infant formulas (1), were purchased from local markets in Western Poland. The empty cans were filled completely with acetonitrile (Super Purity Solvent, Romil Ltd., Cambridge, Great Britain), and the extraction process was carried out by carefully stirring the solution for 90 min. The extracts were concentrated to a minimum by vacuum evaporation (Rotavapor R-120, Büchi, Essen, Germany) and made up to a final volume of 3 mL with acetonitrile. Prior to the HPLC/ESI-MS analysis, the sample was further filtered through syringe filters with a pore size of 0.45 μm (Macherey-Nagel GmbH, Düren, Germany). The presence of BADGE + BuO(EtO)nH + BuO(EtO)mH was identified in the two extracts of empty meat cans. The data presented henceforth were obtained for one of the extracts. The data obtained for the second extract were very similar, which means that most probably the same coating materials were used for the production of these cans.

The HPLC/ESI-MS analyses were made on a Waters model 2690 HPLC pump (Milford, MA, USA), Waters 996 Photodiode Array Detector, and Waters/Micromass ZQ2000 mass spectrometer (a single quadrupole type instrument equipped with an electrospray ion source, Z-spray, Manchester, UK). The software used was MassLynx V3.5 (Manchester, UK). The sample solutions were injected onto the XBridge^®^ C18 column (3.5 µm, 100 mm × 3 mm i.d.; Waters, Warsaw, Poland) using an autosampler. The injection volume was 10 µL. The solutions were analyzed using a linear gradient of CH_3_CN-H_2_O with a flow rate of 0.4 mL/min. The gradient started from 0% CH_3_CN–95% H_2_O with 5% of a 10% solution of formic acid in water, reaching 95% CH_3_CN after 15 min, and the latter concentration was kept for 15 min.

The ESI mass spectra were recorded in the *m*/*z* range 100–1000 in positive ion mode. The electrospray source potentials were: capillary 3 kV, lens 0.5 kV, extractor 4 V, and cone voltage 30–80 V. It is known that cone voltage has the greatest impact on the mass spectra recorded. An increase in this parameter leads to the so-called “in-source” fragmentation/dissociation, but a too low cone voltage may cause a decrease in sensitivity. The source temperature was 120 °C, and the desolvation temperature 300 °C. The nebulizing and desolvation gas were nitrogen at the flow rates of 100 and 300 L/h, respectively.

In order to obtain the accurate masses of the compounds of interest and confirm their elemental composition, we collected the eluates containing the compounds (in the proper range of retention time), and then we directly infused the eluates into the Q-TOF mass spectrometer (coupling off-line). The accurate mass measurements were performed using an Impact HD mass spectrometer (Bruker Daltonics, Bremen, Germany). The collected fractions were diluted ten times in methanol and infused into the ESI source by a syringe pump (direct inlet) at a flow rate of 3 µL/min. The instrument was operated under the following optimized settings: end-plate voltage 500 V; capillary voltage 4.2 kV; nebulizer pressure 0.4 bar; dry gas (nitrogen) temperature 180 °C; dry gas flow rate 4 L/min. The spectrometer was previously calibrated with the standard tune mixture.

## 3. Results

**Figure 1:**
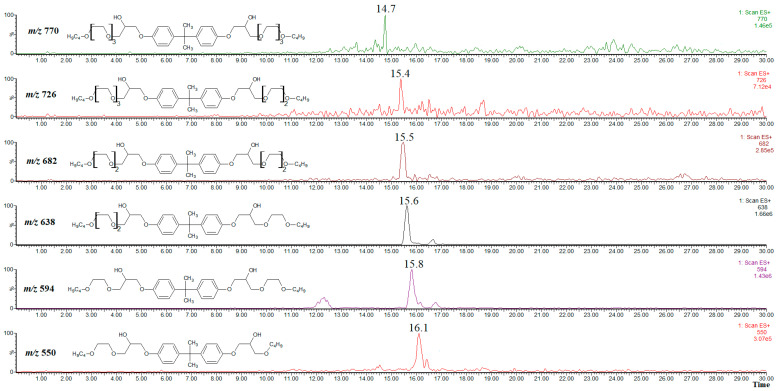
Single ion chromatograms of [M + NH_4_]^+^ ions of ethoxylated butoxyethanol-BADGE adducts.

During an HPLC/ESI-MS analysis, BADGE and similar compounds are usually detected as [M + NH_4_]^+^ ions, even when no ammonium salt is added to the mobile phase [15,16,22]. In our experiment, the ethoxylated butoxyethanol-BADGE adducts were also detected as [M + NH_4_]^+^. Ammonium cations are most probably present in acetonitrile as contaminants, and the formation of [M + NH_4_]^+^ means that molecule M has a relatively high affinity towards NH_4_^+^. Figure 1 shows the single ion chromatograms of [M + NH_4_]^+^ ions. It is clear that the compounds containing more oxyethylene units are eluted first. As the last one, the adduct of BADGE with BuOEtOH and butanol was detected (Figure 1, *m*/*z* 550). This compound contained only one oxyethylene unit. The largest detected adduct was BADGE + 2BuO(EtO)_3_H (*m*/*z* 770), although the observed signal-to-noise ratio was low (Figure 1). On the other hand, the signal-to-noise ratio of ions at *m*/*z* 770 and 726 is above 3; thus, their detection is undisputable. In other words, the concentration of the compounds containing more than 4 oxyethylene units is low, however, still above the detection limit (of course, without pure standards, it is not possible to evaluate the detection limit).

**Figure 2:**
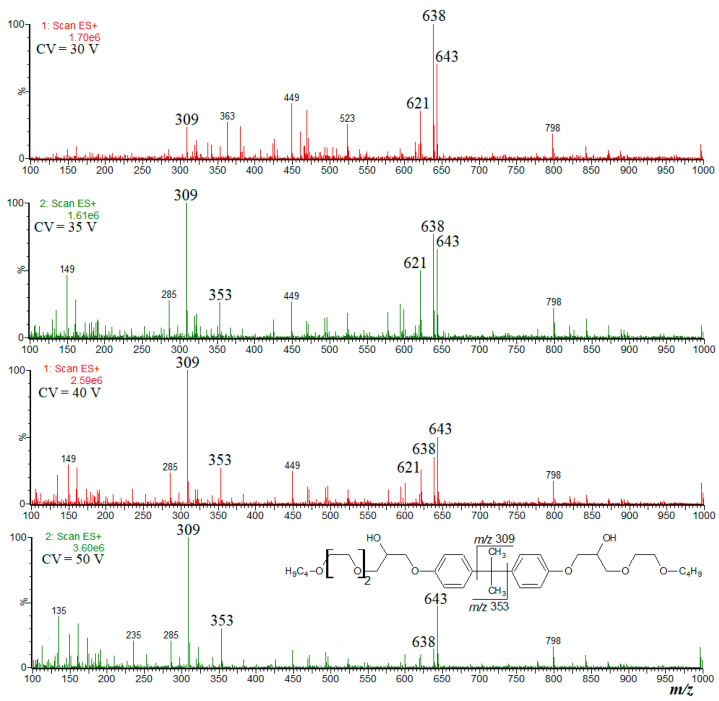
ESI mass spectra of BADGE + BuO(EtO)_2_H + BuOEtOH obtained during HPLC/ESI-MS analysis.

Figure 2 shows the ESI mass spectra of BADGE + BuO(EtO)_2_H + BuOEtOH as representative examples. Although the spectra were obtained during HPLC/ESI-MS analysis, it was not possible to obtain the spectra of pure compounds (of course, we checked if the ions derived from a given compound had identical retention times; otherwise we deal with backgrounds/coeluting (overlapping) compounds). At a cone voltage of 30 V, the most abundant ion was [M + NH_4_]^+^ at *m*/*z* 638, and ions [M + Na]^+^ and [M + H]^+^ at *m*/*z* 643 and 621, respectively, were also clearly seen. At higher cone voltages, the most abundant was the product ion at *m*/*z* 309, while the product ion at *m*/*z* 353 was also clearly seen (Figure 2). The observed fragmentation pathways are analogous to those observed for BADGE and its derivatives and confirm the structure of BADGE + BuO(EtO)_2_H + BuOEtOH [23,24,25].

**Figure 3:**
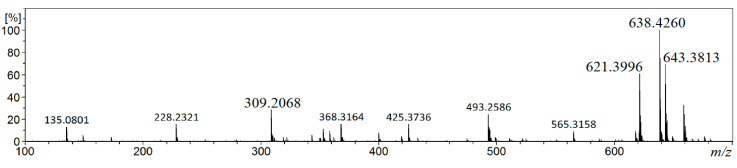
The high-resolution mass spectrum of BADGE + BuO(EtO)_2_H + BuOEtOH.

The ions accurate masses obtained from the high-resolution mass spectra confirmed their elemental compositions, thus the structures of ethoxylated butoxyethanol-BADGE adducts are shown in Table 1 for [M + NH_4_]^+^ ions. Figure 3 shows the high-resolution mass spectrum of BADGE + BuO(EtO)_2_H + BuOEtOH as a representative example.

Besides the adducts of BADGE with ethoxylated butoxyethanols, we also detected other adducts with ethoxylated butoxyethanols namely BADGE + H_2_O + BuO(EtO)_n_H and BADGE + BPA + BuO(EtO)_n_H, however, only the short ones (n = 1, 2), as shown in the supplementary material (Figures S1 and S2). To the best of our knowledge, those with *n* = 2 have not been detected yet in such extracts. Of course, we have also detected a number of other compounds, among them cyclo-di-BADGE (Figure S3), which is the typical compound present in the epoxy resin material [3,4,13,15,16]. 

## 4. Discussion

As mentioned in the experimental section, the discussed adducts were detected in two extracts out of ten. It means that they are not very common; however, common enough to be a matter of study. Furthermore, the detection of the adducts in two extracts of empty meat cans out of three extracts may suggest that in this type of can they are quite common.

The question is, what are the reasons for the presence of the adducts of BADGE with ethoxylated butoxyethanols in the epoxy resin material. One of the reasons, mentioned in the introduction, can be the reaction between BADGE and BuO(EtO)_n_H; the latter is formed in the reaction between butoxyethanol and ethylene oxide. On the other hand, in order to obtain the desired properties of epoxy resin material, it may be modified by incorporation of oxyethylene units, as described in detail elsewhere [26,27,28]. Thus, such a modification can also be a source of ethoxylated species (these detected in this work or others).

The next important challenge is the problem of migration of the detected adducts from can coating material into canned food products. It is expected that the compounds containing fewer oxyethylene units, as more lipophilic, can better migrate into meat products, whereas the compound containing more oxyethylene units, as more hydrophilic, can better migrate into fruit products. Of course, a number of factors can affect the migration process [7,10,12,29]. If the migration ability of the adducts is high, the question is what can be their influence on human health. To the best of our knowledge, until present there have been no biological data concerning detected earlier BADGE adduct with butoxyethanol, thus it is even difficult to speculate about the influence of BADGE adduct with ethoxylated butoxyethanols on human health.

Summing up, in this preliminary study, the presence of the discussed adducts in the extracts of epoxy resin material has been unambiguously proved by using HPLC/ESI-MS, and, as a consequence, a number of detailed studies devoted to the above problems should be made in future.

## Figures and Tables

**Table 1:** Confirmation data of elemental composition of [M + NH_4_]^+^ ions.

Compound	Composition of [M + NH_4_]^+^	Exact Mass	MeasuredMass	Error (ppm)
BADGE + BuOEtOH + butanol	C_31_H_52_O_7_N	550.3744	550.3726	−3.3
BADGE + 2BuOEtOH	C_33_H_56_O_8_N	594.4006	594.3996	−1.7
BADGE + BuOEtOH + BuO(EtO)_2_H	C_35_H_60_O_9_N	638.4268	638.4260	−1.3
BADGE + 2BuO(EtO)_2_H	C_37_H_64_O_10_N	682.4530	682.4524	−0.88
BADGE + BuO(EtO)_2_H + Bu(EtO)_3_H	C_39_H_68_O_11_N	726.4792	726.4777	−2.1
BADGE + 2BuO(EtO)_3_H	C_41_H_72_O_12_N	770.5055	770.5043	−1.6

## Data Availability

Data sharing is not applicable to this article.

## Supplementary Materials

The following are available online at https://www.mdpi.com/article/10.3390/ma14133682/s1, Figure S1: HPLC/ESI-MS detection of BADGE + BuEtOH +H_2_O (M = 476) and BADGE + Bu(EtO)_2_H +H_2_O (M = 520), Figure S2: HPLC/ESI-MS detection of BADGE +BPA + BuEtOH (M = 686) and BADGE +BPA + Bu(EtO)_2_H (M = 730), Figure S3: HPLC/ESI-MS detection of cyclo-di-BADGE (M = 568). Beside the ions [M + H]^+^ and [M + NH4]^+^ (*m*/*z* 569 and 586, respectively) the abundant product ion at *m*/*z* 191 (characteristic for BADGE related compounds, [16]), has been observed.
