# Supplementary material for: Ethoxylated Butoxyethanol-BADGE Adducts—New Potential Migrants from Epoxy Resin Can Coating Material
Source: Materials (Basel). 2021 Jul 1;14(13):3682. doi: 10.3390/ma14133682 (PMC8269880; doi:10.3390/ma14133682)
Supplement: Supplementary file 1 [file materials-14-03682-s001.zip › materials-1241400-supplementary.pdf]

materials

Supplementary Material

Ethoxylated Butoxyethanol-BADGE Adducts—New Potential Migrants from Epoxy Resin Can Coating Material

Monika Beszterda [1], Małgorzata Kasperkowiak [2], Magdalena Frańska [3], Sandra Jęziołowska [4] and Rafał Frański [4,*]

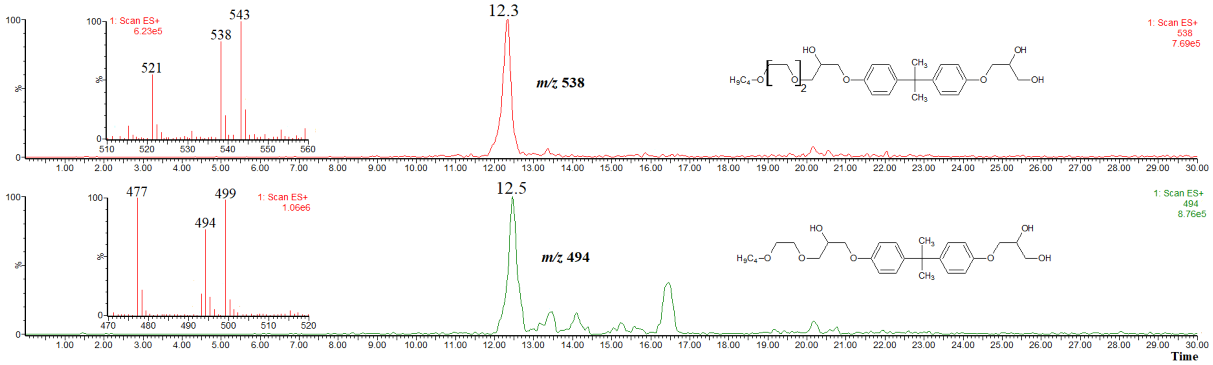

Figure S1. HPLC/ESI-MS detection of BADGE + BuEtOH +H_2O (M=476) and BADGE + $Bu(EtO)_2H$ + H_2O (M=520).

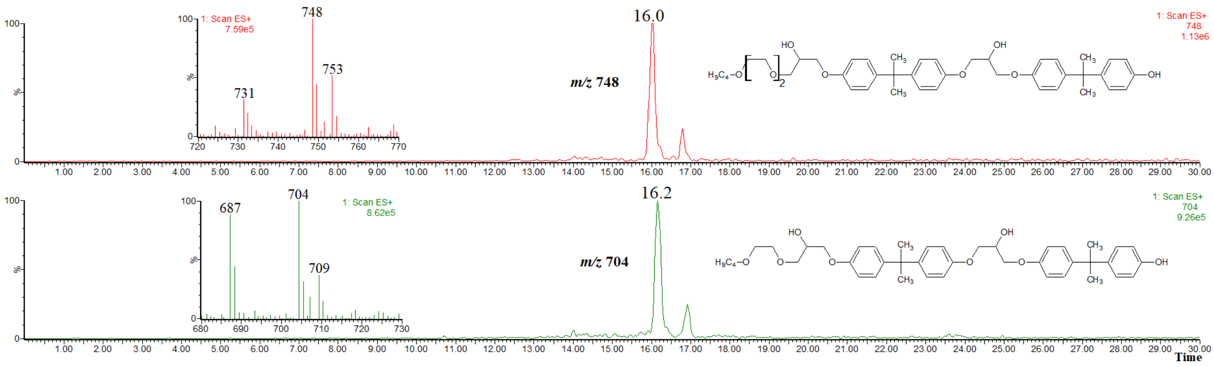

Figure S2. HPLC/ESI-MS detection of BADGE + BPA + BuEtOH (M=686) and BADGE +BPA + $Bu(EtO)_2H$ (M=730).

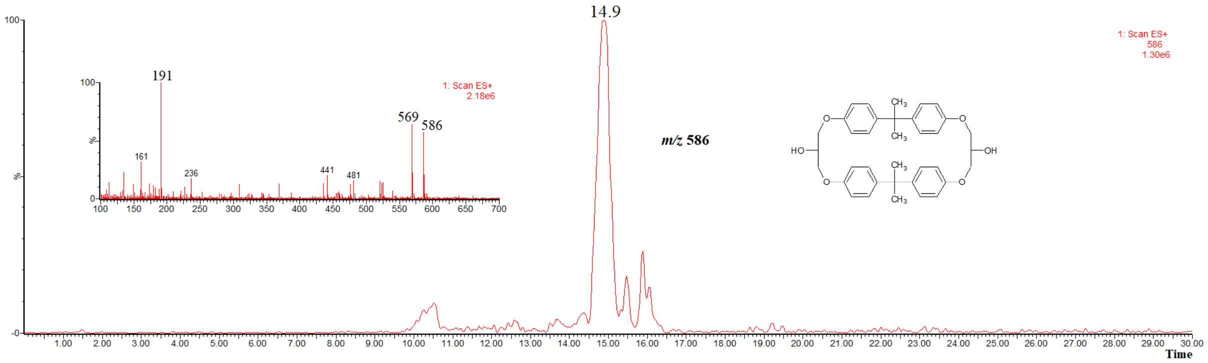

Figure S3. HPLC/ESI-MS detection of cyclo-di-BADGE (M=568). Beside the ions $[M+H]^+$ and $[M+NH_4]^+$ (*m*/*z* 569 and 586, respectively) the abundant product ion at *m*/*z* 191 (characteristic for BADGE related compounds, [16]), has been observed.
